# Low Temperature Plasma: A Novel Focal Therapy for Localized Prostate Cancer?

**DOI:** 10.1155/2014/878319

**Published:** 2014-03-13

**Authors:** Adam M. Hirst, Fiona M. Frame, Norman J. Maitland, Deborah O'Connell

**Affiliations:** ^1^Department of Physics, York Plasma Institute, University of York, Heslington, York YO10 5DD, UK; ^2^YCR Cancer Research Unit, Department of Biology, University of York, Heslington, York YO10 5DD, UK

## Abstract

Despite considerable advances in recent years for the focal treatment of localized prostate cancer, high recurrence rates and detrimental side effects are still a cause for concern. In this review, we compare current focal therapies to a potentially novel approach for the treatment of early onset prostate cancer: low temperature plasma. The rapidly evolving plasma technology has the potential to deliver a wide range of promising medical applications via the delivery of plasma-induced reactive oxygen and nitrogen species. Studies assessing the effect of low temperature plasma on cell lines and xenografts have demonstrated DNA damage leading to apoptosis and reduction in cell viability. However, there have been no studies on prostate cancer, which is an obvious candidate for this novel therapy. We present here the potential of low temperature plasma as a focal therapy for prostate cancer.

## 1. Introduction

Prostate cancer is now recognised as the second most diagnosed cancer overall and accounts for around a quarter of all cancers in males [1]. The risk of prostate cancer peaks in men over 60 years of age, yet high incidence rates are also found in younger aged groups [2]. In addition, benign enlargement of the prostate becomes increasingly common in men over the age of 40 and particularly so beyond 60 years of age [3].

Treatment for advanced prostate cancer is still unsatisfactory, with an almost inevitable development of hormone resistance [4]. Even new generation androgen ablation drugs fail to deliver a life extension beyond several months [5]. In addition, there is poor response to chemotherapy, alongside unpleasant side effects, and reduced quality of life [6]. Therefore, the emphasis remains to detect and treat prostate cancer at an early stage to have most hope of a cure. Indeed, early diagnosis has become more common with increased uptake of PSA testing [7, 8].

Once prostate cancer is diagnosed, the clinician is presented with a series of dilemmas; firstly, is the tumour localized or has it spread [9]; second, if localized is it potentially aggressive or indolent [10]; and the third, should the patient undergo active surveillance or be treated immediately [11]. If the latter is chosen in the context of a localized tumour, then the next decision is between radical surgery with the risk of incontinence and impotence, radiotherapy, or treatment with a focal therapy [12]. Radical surgery has the potential to be an overtreatment in early-onset or low-risk disease [13], where active surveillance or treatment with a focal therapy may be more suitable [14]. Ideally, focal therapy is targeted to maximize elimination of the tumour foci without treating the whole gland, while minimizing side effects [15, 16]. This review aims to evaluate several currently available focal therapies for prostate cancer and introduces a potential focal treatment in the form of low temperature plasma (LTP). Application of LTPs to internal organs such as the prostate may seem technically difficult but could offer many advantages over current treatments.

## 2. Approaches to Focal Therapy of Localized Prostate Cancer

For patients to be considered as candidates for focal therapy, their prostate cancer must be present in only one lobe, typically unifocal, and contained within the prostate capsule [17]. However, no absolute ideal patient selection criteria exist for focal prostate treatment [18]. In the following subsections, some focal therapies for localized prostate cancer are briefly analyzed, with their respective advantages and pitfalls outlined for comparison. In addition, the importance of imaging techniques in the context of focal therapy treatments is also discussed.

### 2.1. High-Intensity Focused Ultrasound

The concept of high-intensity focused ultrasound (HIFU) was first applied in the 1980s to benign prostate hyperplasia (BPH) [19], with the first recorded application to localized prostate cancer in 1995 [20]. The physical mechanism of HIFU follows the same principles as diagnostic ultrasound, whereby ultrasonic waves pass through healthy tissues without causing harm. However, if the ultrasonic beam is sufficiently focused and the intensity increased, high levels of energy can be delivered to very localized regions [7]. These high levels of energy are capable of causing irreversible damage to the targeted tissue via hyperthermia mechanisms, either by heating or inertial cavitation [7, 21, 22]. In the case of thermal effects, energy delivered by the ultrasonic beam is absorbed by the treated area, leading to rapid heating effects, which can raise the temperature of the treated tissue to 80°C in a few seconds [23]. This instant heating leads to coagulative necrosis through protein denaturation [15, 24]. A recent study considered the treated area to have been successfully ablated once a minimum temperature of 65°C had been reached [25].

The typical devices used for HIFU treatment of the prostate are applied transrectally and so possess the advantage over other focal therapies in that an invasive surgical approach is not required. There are two devices currently available for HIFU: Sonablate and Ablatherm. Taking Sonablate as an example, the device utilizes a 4 MHz transducer which is capable of both treatment and imaging depending upon the intensity applied, with intensities of up to 2000 W cm^−2^ achievable at focal lengths as short as 3 cm [26]. Due to the extremely high intensities involved in the procedure, there is a need for accurate monitoring of the energy delivery to, and resulting temperature of, the target tissue. In recent years, the effectiveness of real-time magnetic resonance imaging (MRI) has improved, such that it constitutes an invaluable tool for the monitoring of the HIFU procedure [25, 27].

The difficulty with treating enlarged prostates lies mainly in limitations on the focal length of the ultrasound probe [22, 28]. A transurethral resection of the prostate (TURP) procedure is recommended prior to treatment to reduce organ volume, as post-HIFU swelling of the prostate is common [8, 29]. The effective treatment of anterior prostate tumours is also problematic using HIFU, as anterior perirectal fat tissue can prevent intended penetration depth of the ultrasound beam [30]. This occurs due to reflection of the signal and is a particular problem if the patient is overweight [31].

### 2.2. Photo-Dynamic Therapy

Photodynamic therapy (PDT) damages tissues in a highly localized fashion by exciting photosensitizing drugs with light. The drugs are administered either orally or intravenously, absorb energy from a light source, for example a laser, and transfer it to molecular oxygen residing in the surrounding tissues [32]. This in turn produces an activated form of molecular oxygen [33] known as singlet delta oxygen (^1^O_2_, SDO). It is believed that SDO is predominantly produced following the excitation of the sensitizing agent from its triplet ground state, upon irradiation from the light source [34]. SDO is highly toxic to cells and can interfere with cell signalling as well as inducing cellular stress [35–37]. Importantly, the photosensitizing process is recurrent, eliminating the need for repeated applications during delivery as a stream of SDO is produced [33]. In addition, PDT has the advantage of greater selectivity versus other cancer therapies, as only simultaneous exposure to the photosensitizing drug, light, and oxygen will result in a cytotoxic effect on the treated cells [34]. This selectivity can be further improved by the use of an antibody, applied in conjunction with the photosensitizer, which is specific to the tumour [38, 39].

PDT predominantly utilizes two approaches to damage cancerous tissue. Either tumour hypoxia can be induced following laser targeting of the blood supply to the tumour or an apoptotic/necrotic response can be initiated following direct targeting of the tumour surface itself [40]. It is necessary to protect the skin and eyes of the patient, even following treatment. Such protection may be required for a few hours up to several weeks, depending on the photosensitizer used [41], as the time each drug remains in the patient's bloodstream varies vastly. A transperineal approach allows treatment of tumours localized to anterior prostate [42], giving advantages over other treatment approaches such as HIFU (see Table 1), although this can still be problematic [7]. However, PDT has the advantage of being potentially applicable at the same treatment site multiple times [42], unlike for instance surgery or radiotherapy, in addition to being a potential salvage therapy following failure of these techniques [43].

### 2.3. Cryotherapy

Rapid freezing and thawing cycles are employed by cryotherapy techniques in order to cause localized cellular destruction due to either the extremely low temperature alone, the rapid rate of cooling, or the period of time for which the tissue stays frozen [21]. Either liquid nitrogen or argon gas is administered to the prostate transperineally via cryoprobes under transrectal ultrasound (TRUS) guidance. Argon gas probes are now favoured over liquid nitrogen based approaches due their thinner diameters, permitting the insertion of additional probes (in a brachytherapy-like manner) to improve the efficacy of treatment [44]. In addition, the use of argon gas dramatically improves the freeze-thaw effect by reducing the probe tip to a temperature of −187°C, before 67°C helium gas rapidly thaws the treated region [44, 45], causing rupturing and bursting of the cells. Two cycles, reaching at least −40°C are required for complete cell death, with cell shrinkage and protein denaturation occurring as the tissue temperature decreases beyond 0°C [21]. A urethral warming catheter and multiple thermosensors are typically used to prevent freezing of unwanted regions [45, 46].

Cryotherapy can be applied as a salvage therapy, for example, after the failure of or recurrence following radio- and brachytherapy [47, 48]. Common side effects following cryotherapy include rectal or perineal discomfort [49] and urinary infections [50]. Major complications can include rectourethral fistula, although this is rare [45].

### 2.4. Radiotherapy

Whilst radiotherapy is not considered a focal therapy, variants such as Cyberknife and brachytherapy have the potential to be applied to more localized cancers and are discussed later in this section. It has long been known that ionizing radiation (IR) can lead to adverse effects on cells. Using this principle, effects include, but are not limited to DNA damage, cell cycle arrest, and ultimately cell death can be achieved through radiotherapy [51]. This is due to reactive oxygen species (ROS) formed from interactions with free radicals, produced as a result of multiple ionizations via the Compton effect [52]. Radical formation is believed to take place in discrete regions [51], with so-called “clustered” DNA damage necessary in order to produce a potentially lethal cellular effect [53, 54]. However, it has been shown that cancer stem cells (CSCs), which are thought to instigate cancerous growth [55], can be resistant to radiological techniques, as well as promoting cancer recurrence following treatment [56, 57]. Indeed, prostate stem-like cells in epithelial cultures derived from patient samples are more radioresistant than more differentiated cells, due to increased levels of heterochromatin conferring a protective effect [58].

Some studies have suggested that at least 74 Gy, and indeed upwards of 80 Gy [59], should be applied in the case of localized prostate cancer, as patients treated with less than 72 Gy have shown higher cancer recurrence rates [60]. The total dose is usually delivered in multiple smaller fractions of, for example, 2 Gy per day for 60 days, not including weekends [61]. Following treatment, patients may often experience side effects including but not limited to urinary incontinence, diarrhoea, and rectal discomfort. Urinary problems can persist or present at longer time periods following initial treatment, as well as erectile dysfunction [62, 63]. In addition, and most worryingly, a third of patients experience radiorecurrent disease [64].

Different techniques are available, whereby the radiation is either deposited externally or internally. For the external treatment of tumours, the most applied therapy is external beam radiotherapy (EBRT), where the cancerous area is treated by a focused beam of IR. This relies on precise beam alignment with the targeted area, in order to maximize treatment efficacy and minimize collateral damage to surrounding healthy tissue. Several variants of EBRT are being pursued and constantly developed, including three-dimensional conformal radiotherapy (3D-CRT) and intensity-modulated radiation therapy (IMRT), which aim to utilize improvements in imaging technology to satisfy the aforementioned criteria for most effective treatment [65].

Other approaches for the radiological treatment of prostate cancer exist, which rely on the underlying principles of IR, including proton beam therapy. Proton beam therapy has the advantage that protons deliver their energy at the end of the particle's path in the tissue compared to photons which deliver radiation along their path in the tissue [66]. The focal nature of the energy delivery in proton beam therapy could in theory mean that untargeted areas are left unharmed [67]. However, a recent study indicated that damage to irradiated tissues outside of the target area is less severe following IMRT [68], in addition to being of lower cost than proton beam therapy. As such, questions still remain as to the efficacy and effectiveness of proton beam therapy as a focal technique.

Another recent development, which seeks to improve localization of radiotherapy compared to EBRT, is hypofractionated stereotactic body radiation therapy (SBRT) via the Cyberknife linear accelerator machine. A unique feature of prostate cancer is its low “*α*/*β* ratio,” which represents nonrepairable versus repairable cellular damage,respectively, with the *α*-term linearly dependent on administered dose and the *β*-term to its square [69]. For this reason several studies have suggested that hypofractionated radiation doses may result in more effective treatment of localized tumours [69–72]. During Cyberknife SBRT movement of the prostate is detected and automatically corrected for during the procedure by the robotic arm [73], enabling delivery of the radiation to be directed within 2 mm of the target area [74]. This enables the Cyberknife to deliver a hypofractionated radiation dose more accurately and noninvasively to the tumour [73] than conventional EBRT. Another major advantage of SBRT over EBRT is that treatments are usually delivered over a few days rather than weeks, rendering posttreatment hospitalization unnecessary [73]. However, SBRT treatment results in similar side-effects to those experienced following conventional radiotherapy. Rectal and urinary complications have been reported, in addition to erectile dysfunction [70], although the levels of these have been proposed as within acceptable limits [75]. In addition, the cost of Cyberknife technology is more expensive than other radiological techniques, at least in terms of initial outlay [72], although this is yet to be thoroughly investigated.

An increasingly common approach for treating prostate cancer internally is brachytherapy, which uses radioisotopes such as ^125^I, ^103^Pd, and ^131^Cs and is typically applied in order to ablate the whole prostate gland [76]. The radioisotopes, with half-lives ranging from ~10–60 days [76] are delivered to the prostate as seeds through a matrix of narrow diameter needles inserted transperineally. Brachytherapy can either be used as a stand-alone treatment, in conjunction with radiotherapy or radical prostatectomy, or as a salvage treatment following EBRT [77].

A more recent development of brachytherapy is known as high dose rate (HDR) brachytherapy with ^192^Ir [76], which provides a boosted dose of radiation following EBRT [78]. If administered in conjunction with utilizing imaging tools such as MRI or TRUS, radioactive seeds may be delivered to the targeted area more accurately, providing a case for HDR brachytherapy as a focal therapy [79].

### 2.5. Imaging: An Integral Part of Focal Therapy

All focal therapies for prostate cancer rely on accurate imaging to have maximum effect [79]. Imaging techniques are constantly advancing and are used for initial detection, determination of tumour location, staging of tumour, assessment of tumour aggressiveness, and detection of recurrences as well as identifying metastases [80, 81]. In the context of administration of focal therapies, early detection is most critical. Since the widespread uptake of the PSA test, early detection has become more common [79].

The kinds of imaging used in prostate cancer detection, diagnosis, and treatment are TRUS to enable targeted biopsies; MRI for accurate imaging of soft tissue; positron emission tomography (PET) for detecting lymph node metastasis and bone scans; and X-rays and computerized tomography (CT) scans to assess bone metastases. To detect localized prostate cancer, TRUS and MRI are by far the most used and most useful scans.

TRUS was traditionally used to allow biopsies from predetermined sites in the prostate, following an abnormal digital rectal examination (DRE) and increased PSA, and not as a method to identify precise locations of tumour foci [82]. However, improvements to the technique, including contrast-enhanced ultrasound using microbubble contrast media, elastography to measure tissue stiffness, and Doppler ultrasound to measure blood flow, can result in more targeted biopsies, leading to improved detection and diagnosis [83, 84]. TRUS can distinguish between an outer and inner gland encompassing the central, peripheral, and transition zones, though not at the same resolution as MRI [82].

MRI is highly sensitive and is the dominant imaging modality used for focal treatment [85, 86]. To undertake standard MRI, endorectal and pelvic phase-arrayed coils are used in conjunction to improve positioning of the prostate and to receive MR signals, respectively, resulting in clearer images with optimal signal-to-noise ratio [79]. The prostate zones are clearly visualized using MRI [87]. However, standard MRI is not accurate enough to determine precise location and diagnosis, where multiparametric MRI is required [88]. This includes diffusion-weighted imaging (DWI-MRI) that measures water diffusivity, dynamic contrast enhanced (DCE-MRI), making use of a contrast agent, and proton magnetic resonance spectroscopic imaging (H MRSI) that measures metabolites (citrate, choline, creatine, and polyamines), the ratios of which change between normal and cancerous prostate [89]. The technology to allow real-time MRI-guided biopsy has also advanced, and it is conceivable that this would be the ultimate method used when administering any focal therapy, including low temperature plasma-based treatment [88, 90–92].

In order for the more sophisticated imaging procedures to become routine, there has to be access to specialized equipment and personnel (e.g., magnetic resonance physicists), which both contribute to the potentially prohibitive expense [93]. To confidently choose focal therapy as a treatment option, patients and clinicians alike have to be convinced of its effectiveness. Imaging technology and both present and future focal therapy procedures therefore need to evolve in tandem to assure focal tumour ablation.

## 3. Low-Temperature Plasmas and Their Use in Biomedicine

Low temperature plasmas are emerging as an exciting development for therapeutics. The unique properties of cold nonequilibrium plasmas have enormous potential in disease therapeutics and plasma pharmacology as drug alternatives. Applications of these plasmas range from surface sterilization and bacterial decontamination [94–99], biofilm inactivation [100–102], antimicrobial treatment in food preservation [103–105], and wound healing [106, 107], to cancer treatment [108–111]. This rapidly growing field of “plasma medicine” has emerged over the last 5–10 years and offers great potential, bringing together multidisciplinary branches of science and engineering.

Nonequilibrium plasmas, operated at ambient atmospheric pressure and temperature, are very efficient sources for the production of highly reactive neutral particles, for example, reactive oxygen and nitrogen species (RONS) (such as atomic oxygen [112–114], atomic nitrogen [115], hydroxyl radical, superoxide, singlet delta oxygen, and nitrogen oxides), charged particles, UV-radiation, and electromagnetic fields. Individually, many of these components have been implicated in therapeutics. RONS are known to play a crucial role in biological systems, such as signalling and generating oxidative damage to a variety of cellular components, which can ultimately lead to cell death [116–119]. Graves presents a comprehensive review summary on the role of RONS of relevance for plasma applications in biology [120]. Plasmas have the advantage of delivering these* simultaneously*, providing potentially superior processes. The role of these plasma components, even individually, is to date not fully known and is a topic of current research. It can be anticipated that, similar to low pressure plasma processes, in for example, plasma etching or plasma deposition, synergistic mechanisms govern the plasma surface interface rather than the individual species themselves.

### 3.1. Methods of Plasma Formation and Production of Reactive Species

The low temperature plasma environment is actually quite remarkable. Plasmas are formed by applying a sufficiently high electric field across a region of gas such that electrons are stripped off atoms and breakdown of the gas occurs. These free electrons in the background gas are accelerated by the applied field and collide with ions and neutral gas molecules through various processes, which are discussed below. An important feature is that the electrons are not in thermodynamic equilibrium with the background gas due to the largely different masses (light electrons, heavy atoms, and molecules). The background gas is the dominating constituent and is at room temperature, while the electrons are hotter and can drive a unique reactive environment. Ions and electrons can be created through ionization, and processes such as excitation and dissociation of the background gas result in, for example, formation of metastable particles, reactive species, radicals, and also radiation. These plasmas essentially create an otherwise impossible dry, chemically reactive environment at room temperature. Until recently, atmospheric pressure plasmas have been unstable and low temperature plasmas have conventionally been operated under lower gas pressure conditions. While this approach has proven extremely beneficial, for example, in the multibillion dollar semiconductor industry, it is limiting with regard to broader exploitation of nonvacuum compatible materials. Through the use of gas flow it is now possible to sustain stable, controllable plasmas at atmospheric pressure. Reactive species can be brought from the main plasma production region, transporting energy to a surface. Here, two distinctly different plasma sources, with varying degrees of reactivity will be discussed.

**Figure 1:**
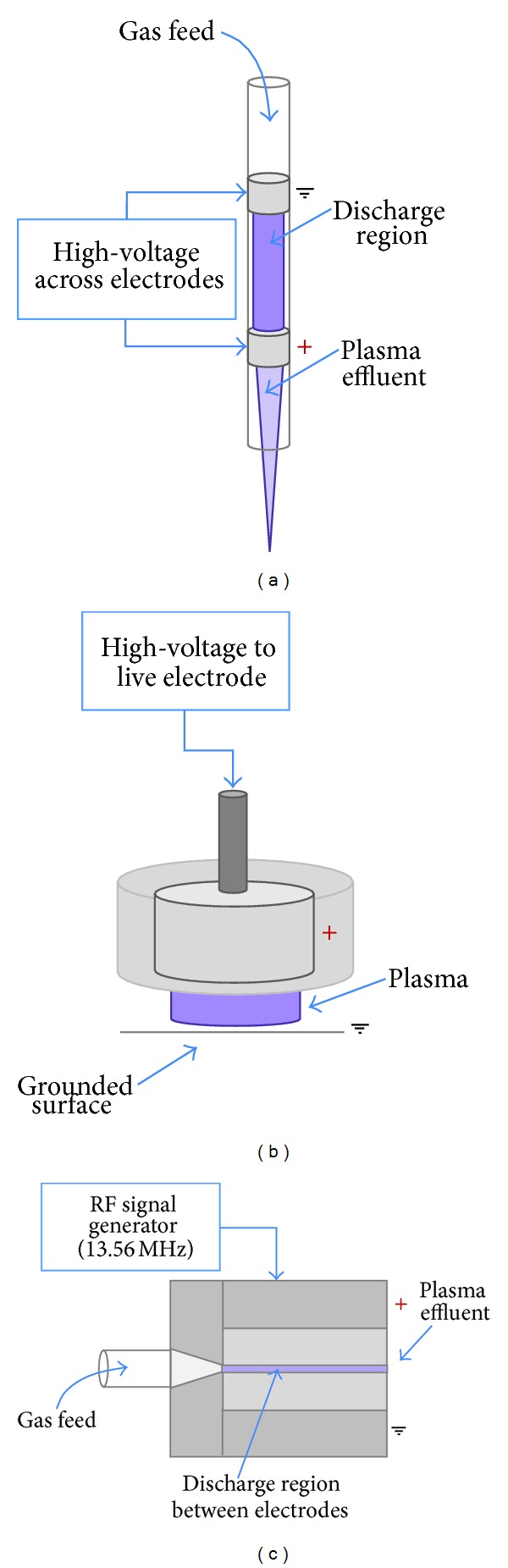
Examples of different plasma devices for medical applications. Linear-field plasma jets: (a) dielectric barrier discharge jet configuration (DBD), (b) floating-electrode DBD (FE-DBD), and cross-field plasma jets (c) radiofrequency (RF).

**Figure 2:**
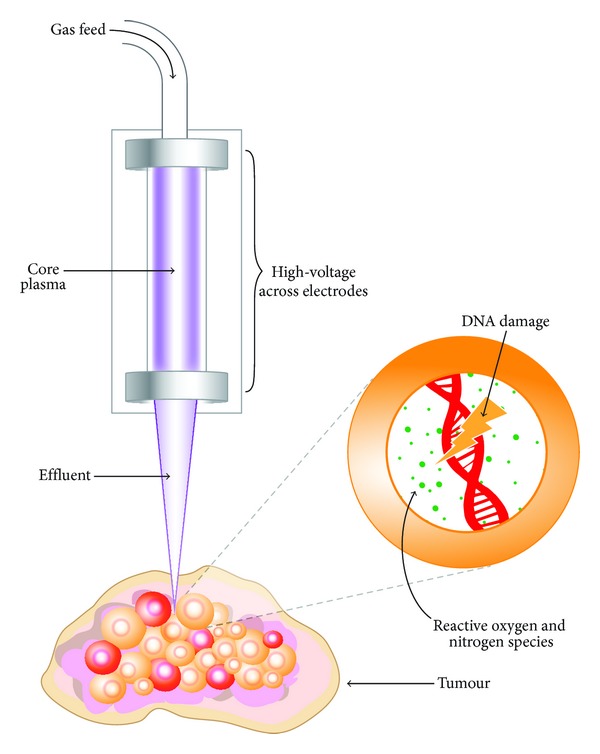
Illustrative diagram conveying the interaction of a DBD plasma jet with a cancerous tumour, leading to the induction of intracellular RONS, DNA damage, and resultant effects such as cell cycle arrest, cell death, and decreased viability.

Various devices are available for the formation and delivery of plasma [120–124] which rely on broadly the same principles. One variant is the dielectric barrier discharge (DBD) configuration plasma jet (Figure 1(a)). Such plasmas are produced by feeding carrier gas (e.g., helium or argon) with small oxygen admixtures (around 0.5% is typical), across a high voltage kHz operated supply, typically 5–30 kV, generating a discharge between two electrodes of dielectric material. Using helium as a buffer gas provides a flexible parameter space for stable homogenous operation at cold gas temperatures. The resulting plasma plume self-propagates outwards, and as the dynamic high electric field is parallel to the direction of propagation, the jet contains reactive neutrals, charged particles, electric fields, and UV radiation (Figure 2). A variation of the DBD schematic is the floating electrode dielectric barrier discharge (FE-DBD) plasma (Figure 1(b)), which operates by using the surface to which it is applied as a floating counter electrode. This is possible provided that the surface has sufficient “charge storage” [94]. FE-DBD has even been applied to human skin without causing thermal damage or unwanted effects [125].

A third example of a plasma source arrangement is the radiofrequency (RF), or cross-field, plasma source (Figure 1(c)) which uses a 13.56 MHz RF signal as a means of gas excitation. This device utilizes plane-parallel electrodes, with a gas flow passing through the core plasma volume. This particular source, unlike the DBD plasma jet, possesses a charge-free effluent since the applied electric field is perpendicular to the direction of gas-travel, thus confining charged species to the core plasma region. Due to the high collision frequency at atmospheric pressure, the effluent is devoid of charge carriers and its characteristics are dominated by energy carrying reactive neutrals. The RF plasma jet is the most comprehensively studied LTP with respect to diagnostics and modelling [112, 115, 126–136] and is currently being developed into a reference source.

The transport of the plasma components to the targeted area is complex. In the core plasma region a large, but defined, number of species can be created (including O, N, NO, and O_2_^−^). As the plasma crosses the interface with ambient air, new reactions and components are formed. Upon interaction with either humidity or liquid layers on biological samples (Figure 2) new species with varying lifetimes can be created (OH, H, H_2_O_2_, and ONOO^−^). Energy dissipation at these interfaces is important and to date unclear. Measurements and simulations under this atmospheric pressure environment are challenging, primarily due to the multiphase (solid, liquid, gas, and plasma), strongly nonequilibrium environment with large gradients (e.g., in electric field), high collisionality thus short-lived species and micron length scales. This requires the development of many new techniques for both measurements and models. The plasma chemistry can be deliberately manipulated or optimized for a desired result by fine alterations to gas admixtures or the electron energy distribution function (EEDF) [129, 137]. Despite the multitude of work that has been conducted to diagnose and characterize the RONS produced [126–128] in addition to the ionization processes and mechanisms that occur in LTPs, these are not yet comprehensively understood.

### 3.2. Supporting Evidence for LTP as a Therapeutic Medical Device

As already mentioned, the potential of LTPs has been explored in many different medical areas. One highly active division of research is in the area of bacterial inactivation and surface sterilization. It has been shown that LTPs can damage the membranes of bacterial cells, through the interaction of reactive species, leading to the bactericidal effect [138]. Survival has been shown to be greatly reduced following LTP treatment, with a clearly defined voided region forming on the irradiated surface [139], suggesting that the interaction is mediated by short-lived reactive species [140, 141]. LTPs do not cause thermal or chemical damage to the treated surface, presenting an advantage over conventional sterilization techniques [140, 142]. Furthermore, LTPs have also been shown to be effective in the treatment of biofilms, minimizing bacterial formation posttreatment [101, 143] and greatly reducing cell viability even at short plasma-exposures [144]. These attributes give potential for applications in dentistry [145, 146].

Plasma-based bacterial inactivation has also been applied to the sterilization of chronic wounds in order to improve the rate of healing. This was shown in a recent phase II trial, which reported a significant reduction of bacterial load in the plasma treated area versus control [147, 148]. Crucially, plasma effects were localized, with no side effects (such as pain due to plasma application) reported. Another trial provided further agreement that LTP does not damage the surface of skin nor lead to dryness through exposure, with a view to antimicrobial applications [149]. It has also been found that when LTP was applied to surface wounds on the skin of mice, vastly improved blood coagulation and consequential accelerated healing resulted versus untreated wounds [106]. It was perceived that plasma-produced NO may be responsible for the improved wound response, which is in agreement with other work on NO as the key RONS in cell proliferation and wound healing [150–154]. Another study showed improved clotting of wounds on the surface of pig skin, in addition to establishing safe operating parameters for LTP exposure [107].

Despite LTPs being earmarked as a technology for future healthcare, plasmas have been used for a range of surgical applications in the field of electrosurgery since as long ago as 1991 [155, 156]. Though not technically a LTP, the argon plasma coagulator (APC) has been employed in various surgical disciplines for the purposes of tissue removal and wound cauterization [155] and is perceived as an improvement on existing laser-based techniques [157]. Recently, plasma vaporization has been applied to benign prostate hyperplasia (BPH), with the hope of reducing the common side effects of conventional transurethral resection of the prostate (TURP) procedures [158]. Early results show that the concept of plasma vaporization could prove to be a significant improvement over current TURP techniques [159] for BPH, with reduced complications [160].

In recent years, investigations have been performed into the interaction of LTPs with different types of cancer cells, including melanoma [161–163], ovarian [164], colorectal [165], liver [166], lung [110], breast [167], and brain [168] amongst others. The gold standard for LTP as a cancer therapy has to be the selective cytocidal targeting of cancerous tissue, whilst leaving healthy tissues unaffected. The effect of reactive species produced by plasma treatment has been extensively studied* in vitro*, with plasma induced DNA damage and apoptosis has been investigated [108, 169]. Another investigation showed the same response due to cellular detachment [170]. A considerable reduction in cell viability has been demonstrated using the MTT assay, as a result of high nitric NO and ROS concentrations [109]. It has also been suggested that immediate cell death can be caused by sufficiently high plasma doses, following minimal cell survival after extended plasma exposure [171]. LTP has also been applied* in vivo* to treat mice with tumours derived from glioma cell lines, where a preliminary study showed a reduction in tumour volume of over 50% at six days following initial plasma treatment [172]. Survival rates of plasma-treated mice increased by over half, compared with the control group who received no treatment [172]. In a follow-up study, ROS produced by the plasma were earmarked as the main antitumour agents, with evidence for cell cycle targeting [173] and apoptosis also presented [174–176]. LTP has also been recently applied to ablate tumours in mice subcutaneously injected with neuroblastoma cells, with a reduction in the rate of tumour growth observed versus control. In addition, survival time posttreatment almost doubled [177]. Another means of LTP-cellular interaction is the electric field that is generated at the effluent tip of DBD jet devices. This may lead to the phenomenon of irreversible cellular electroporation, which has been shown to cause tumour regression and cell death in its own right [178–180] and may aid the transport of RONS to the cell nucleus.

Despite LTP-based approaches demonstrating considerable promise in cancer treatment, further focussed work on the exact mechanisms of plasma-cellular interaction is required before such a technique could be used therapeutically. This includes primarily the quantification of which reactive species are causing adverse cellular effects, tailoring the plasma to deliver maximum damage to the cancer, before developing practical apparatus for patient treatment in the operating theatre. This process may be aided by the use of plasma in combination with radiological and chemotherapeutic techniques [181], in order to increase efficacy.

## 4. Low-Temperature Plasma as a Focal Therapy for Prostate Cancer

At the time of writing, no published study exists on the application of LTP to prostate cancer. The following section outlines how LTPs could be utilized as a focal therapy in practice, how LTPs might compare to other conventional focal therapies for prostate cancer in terms of efficacy, and what might be the upper limit of disease stage for treatment of localized cancer with LTP.

### 4.1. Methods of Treatment Delivery and Plasma “Dose”

**Figure 3:**
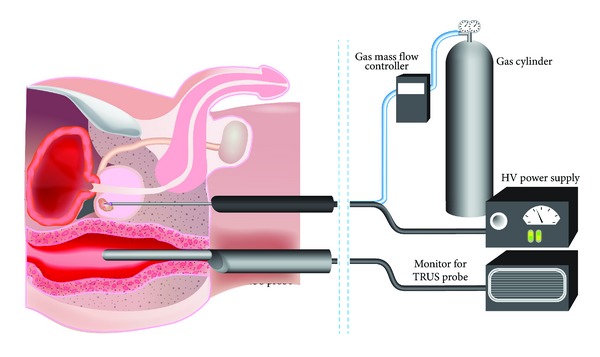
Proposed treatment approach for LTP treatment of localized prostate cancer. The LTP device is administered transperineally to an organ confined prostate cancer. Supporting image guidance from a TRUS probe, along with high-voltage (HV) power supply and gas flow-rate control are shown.

Application of LTP to a patient has been successfully applied clinically by treatment of the skin with the FE-DBD system mentioned earlier [125]. Clearly, delivery to the prostate represents a more complex technical challenge. Probably the most efficient means of application would be to follow the approach of PDT and brachytherapy by inserting the plasma transperineally to provide a focal treatment of organ confined cancers. In reality, whichever way LTP is applied, there is an obvious common dependence on accurate imaging techniques, as discussed in Section 2.5. With simultaneous image guidance by means of TRUS, following prior MRI scan, it is conceivable that LTP could be applied to localized tumours. A representation of one potential treatment delivery system is outlined in Figure 3.

DBD configuration plasma jet devices have already been fabricated and delivered via flexible optical fibres for the treatment of carcinoma, with outer diameters as fine as 60 *μ*m [110].

One of the most important factors for LTP as a cancer therapy is a thorough understanding of the species produced and their concentration for each particular type of device. Correlating the concentration of produced species to a known plasma “dose” is crucial, as lower doses and exposures can stimulate a proliferative response in cells [182, 183]. For LTP therapy to be accepted clinically, there first needs to be agreement on what constitutes the units of plasma “dose.” At present, independent research groups use different devices with different operating parameters (such as those outlined in Figure 1, amongst others), with varying exposure times. Such an agreement would lead to directly comparable data across institutions, which may accelerate the route to the clinic, and thus the patient.

### 4.2. Proposed Efficacy as Compared to Other Therapies

Given that plasma induces ROS, one obvious comparison to current cancer therapies is with radiotherapy, in that both are forms of ionizing radiation that produce reactive species. Besides the lack of a need for radioactive materials, another advantage that LTP possesses over radiotherapy is the production of reactive nitrogen species (RNS) in addition to ROS. As mentioned in Section 3.2, high concentrations of NO have been shown to have a considerable detrimental effect on cell viability, induce apoptosis [184], and have the potential to cause cytostasis in tumour cells [185]. In addition, the production of peroxynitrite (ONOO^−^) formed as a result of reactions between superoxide (O_2_^−^) and NO has been shown to cause DNA damage and oxidation of proteins [186, 187]. Some recent diagnostic studies have demonstrated the production of the radical singlet-delta oxygen by LTPs [188–190], which suggests similarities between LTP treatment and PDT. LTP, however, has the advantage of SDO production in addition to a range of other reactive species with cytotoxic effects.

There is some evidence to suggest that LTP may offer a selective kill of cancerous cells [164, 191–193], which offers a potential advantage over conventional radiological techniques, where unwanted damage to surrounding tissues is the main concern. However, this selectivity is yet to be definitively proven. Furthermore, due to the ambient temperature of the plasma, there should be no requirement for the probes employed by cryoablation (which monitor and regulate the temperature of the urethra and bladder), as thermal effects to the neighbouring tissues should not be of concern. This could offer a more simplified treatment procedure, targeting the tumour bed preferentially.

### 4.3. When to Treat with LTP?

In terms of patient selection for treatment with LTP, similar criteria to current focal therapies would be applied [194]. Patients with low risk cancer (Gleason 6) are likely to opt for active surveillance to avoid unnecessary invasive procedures [195]. Patients with metastatic or locally advanced prostate cancer (typically Gleason 8–10) are not generally considered for focal therapy, as stated in Section 2. Therefore, the final group with intermediate risk prostate cancer would be candidates for LTP therapy. These patients are likely to have a predicted life expectancy of more than five years, with no detection of locally advanced disease using imaging technologies (clinical stage T2a or lower) [43, 196]. Their cancer is likely to be Gleason 7 (although some localized cancer could be Gleason 8) and their PSA should be low (less than 10–20 ng/mL). The other consideration for treatment is whether the tumour is unifocal or multifocal, thereby perhaps necessitating more than one treatment probe. 3D mapping of biopsies should assist in identification of the location, number, and size of tumour foci [197]. Fewer well-defined tumour foci would be logistically easier to treat than multiple foci. Ultimately, such focal therapy treatment is a good option for patients who do not like the uncertainty of watchful waiting but do not want to suffer the side effects of aggressive overtreatment for a low risk cancer.

## 5. Conclusions

In this review we have analysed some of the currently available focal therapies for localized prostate cancer and where their advantages and limitations lie. We propose that the emerging field of low temperature plasmas may offer an alternative and viable solution to the effective treatment of prostate cancer, with minimal side effects and improved treatment efficacy versus other focal therapies. However, for this promising concept to become a reality, further study must be undertaken in order to fully diagnose the cellular interaction mechanisms of the plasma, and also how surgical administration would occur, a means of which has been suggested here. In addition, there is a need for continued development of imaging diagnostics, upon which a plasma-based approach would rely for precise application.

## Figures and Tables

**Table 1:** Pros and cons of focal therapies currently available for prostate cancer.

Treatment	Summary of Pros	Summary of Cons
High-intensity focused ultrasound	(i) Transrectal application negates the need for surgical approach (ii) Improvements in MRI technology allow real-time procedure monitoring and improved targeting	(i) Difficulty treating enlarged prostates, especially in overweight patients (ii) Effective treatment of anterior tumours is not achievable

Photodynamic therapy	(i) More selective than other focal therapies due to conditions needed for SDO production (ii) Can be applied at the same treatment site multiple times	Photosensitizing agent remains in patient's bloodstream following treatment, requiring protection of the eyes and skin for potentially weeks after the procedure

Cryotherapy	(i) Double freeze-thaw cycle effectively destroys cells in targeted region (ii) Can be applied as a salvage following radiotherapy techniques	(i) Urinary infections and perineal discomfort posttreatment are common (ii) Relatively invasive treatment, with added needed for thermal protection of urethra, bladder and rectum

Radiotherapy	(i) Minimally invasive approach as radiation is usually applied externally (ii) Proton beam therapy and Cyberknife technologies give hope of improved targeting with fewer side effects	(i) Many side effects as a result of radiation at unintended sites, causing urinary incontinence, rectal pain, and erectile dysfunction (ii) A third of patients experience radiorecurrent disease

Brachytherapy	Image guided seed placement allows effective treatment of localized areas	Needle array application is a highly invasive process
